# Generic GLP-1 receptor agonists in India: an opportunity to close the cardiovascular evidence gap

**DOI:** 10.1016/j.lansea.2026.100880

**Published:** 2026-10-09

**Authors:** Mohammad Riashad Monjur, Ahmad Alam

**Affiliations:** a Camden Metabolic Rehabilitation Program, South Western Sydney Local Health District, Campbelltown, New South Wales, Australia; b Department of Endocrinology, Rajiv Gandhi Centre for Diabetes and Endocrinology, Jawaharlal Nehru Medical College, Aligarh Muslim University, Aligarh, Uttar Pradesh, India

The semaglutide patent expiry in India in March 2026 is expected to accelerate the entry of generic injectable formulations into a market with more than 100 million adults living with type 2 diabetes.^1^ However, an important evidence gap remains: no cardiovascular outcomes trial of a GLP-1 receptor agonist has specifically enrolled South Asians with sufficient representation for dedicated analysis.

The major injectable semaglutide cardiovascular outcomes trials across diabetes and obesity populations enrolled predominantly Caucasian participants: SUSTAIN-6 (3297 participants; 83.0% Caucasian, 8.3% Asian) and SELECT (17,605 participants; 84.0% Caucasian, 8.2% Asian). Both trials reported race using broad a “Asian” category without disaggregating participants of Indian or South Asian origin, limiting assessment of the generalisability of their cardiovascular benefits to the Indian population.^2^ This concern is clinically important.

Compared to the populations in which these trials were predominantly conducted, the Indian population carries a higher burden of cardiometabolic risk and is more likely to develop atherosclerotic cardiovascular disease and type 2 diabetes at lower body mass index thresholds.^3^ Additionally, this population also exhibit a distinct obesity phenotype, characterised by greater visceral and ectopic adiposity, more pronounced insulin resistance, and a heightened susceptibility to sarcopenic obesity.^4^ At present, the adoption of GLP-1 receptor agonists in India remains limited, largely due to cost and limited specialist infrastructure.^5^ The availability of affordable generic semaglutide is therefore likely to expand the treated population substantially over the coming years, creating a cohort of a scale not previously captured in prospective trials.^1^

This inflection point presents an opportunity to build much needed cardiovascular effectiveness data for India, the broader South Asian region, and the global diaspora. A coordinated research agenda should address whether the cardiovascular benefit of GLP-1 receptor agonists is generalisable to the Indian population, whether cardiovascular risk reduction should be initiated at population-specific BMI thresholds, and what constitutes the optimal titration and maintenance dosing strategy in routine practice. Realising this opportunity will require coordinated investment from health authorities, academic institutions, and industry in prospective multicentre registries across the region, robust regulatory oversight to ensure quality and comparability of peptide generics, and the development of multidisciplinary cardiometabolic care pathways. The window for prospective data capture from the point of market entry is narrow, and once it closes, the evidence gap may only widen.

## Contributors

MRM and AA equally contributed to the correspondence, both authors reviewed and approved the final manuscript.

## Declaration of interests

The authors have no competing interests to declare.
